# High-Sensitivity SWIR Photodetector Based on PbS Quantum Dots via Solution-Phase MAPI Ligand Exchange

**DOI:** 10.3390/s26113391

**Published:** 2026-05-27

**Authors:** Yuntae Ha, JinBeom Kwon, Saewan Kim, Dong Geon Jung, Daewoong Jung

**Affiliations:** 1 Advanced Mobility System Group, Korea Institute of Industrial Technology (KITECH), Daegu 42994, Republic of Korea; hayt223@kitech.re.kr (Y.H.); jdg8609@kitech.re.kr (D.G.J.); 2 School of Electronic and Electrical Engineering, Kyungpook National University, Daegu 41566, Republic of Korea; 3 Department of Semiconductor Engineering, Kyungwoon University, 730, Gangdong-ro, Sandong-eup, Gumi 39160, Republic of Korea; jinbum0301@ikw.ac.kr; 4 Advanced Semiconductor Research Center, IT Materials & Components Research Division, Gumi Electronics & Information Technology Research Institute (GERI), Gumi 39171, Republic of Korea; saewan.kim@geri.re.kr; 5 Department of Nanomechatronics Engineering, College of Nanoscience & Nanotechnology, Pusan National University, 2 Busandaehak-ro, Busan 46241, Republic of Korea

**Keywords:** PbS QDs, SWIR, photodetector, LIDAR, quantum dots

## Abstract

The use of LIDAR sensors is rapidly increasing across various fields, including autonomous transportation, robotics, security systems, and biosensing. Among the core components of LIDAR systems, short-wave infrared (SWIR) sensors play a crucial role in detecting infrared light reflected from objects to recognize the surrounding environment and humans. Various types of SWIR sensors have been reported, with growing demand for those capable of detecting eye-safe infrared wavelengths above 1400 nm. In particular, quantum dot (QD)-based SWIR sensors are attracting attention due to their tunable wavelength range within the eye-safe region, narrow full width at half maximum (FWHM), and selective detection with minimal interference from other infrared wavelengths. Moreover, QD-based SWIR photodetectors can be synthesized and fabricated via solution-based methods, offering advantages such as low cost and ease of fabrication. However, the long organic ligands typically present on QDs exhibit insulating properties, limiting the sensitivity and stability of the photodetectors. To address this issue, organic ligands can be replaced with short inorganic ligands possessing superior electrical conductivity. In this study, the organic ligands of synthesized PbS QDs were replaced with the inorganic ligand methylammonium lead iodide (MAPI) in solution, and a SWIR photodetector was fabricated. The MAPI-capped PbS QD-based photodetector exhibited remarkable external quantum efficiency (EQE) of 62%, a responsivity of 0.73 A/W, and a detectivity of 2.26 × 10^11^ Jones within the 1400–1500 nm wavelength range.

## 1. Introduction

Short-wave infrared (SWIR) photodetectors have attracted significant attention as key sensing components for a wide range of applications, including light detection and ranging (LIDAR), optical communication, industrial monitoring, and autonomous systems. In particular, SWIR detection in the wavelength region beyond 1400 nm is considered eye-safe, making it highly suitable for human-interactive sensing platforms such as automotive and robotic LIDAR systems. As a result, the development of sensitive, stable, and cost-effective SWIR photodetectors has become an important research topic in sensor engineering [1,2]. Conventional SWIR photodetectors based on III–V compound semiconductors, such as InGaAs, InSb, HgCdTe, and HgTe, exhibit excellent sensitivity and low noise characteristics. However, their widespread adoption is hindered by high fabrication costs, complex epitaxial growth processes, and limited scalability. These limitations motivate the exploration of alternative material systems that enable low-cost, solution-processable SWIR sensors while maintaining competitive optoelectronic performance [3,4,5,6,7]. Colloidal quantum dots (QDs), particularly lead sulfide (PbS) QDs, have emerged as promising candidates for SWIR photodetectors due to their size-tunable absorption in the 1000–2800 nm range, narrow spectral bandwidth, and compatibility with solution-based fabrication processes. These characteristics allow PbS QDs to be integrated into flexible and large-area sensor platforms. Despite these advantages, the performance of PbS QD-based SWIR photodetectors is often limited by the presence of long-chain organic ligands, such as oleylamine, which act as insulating barriers and restrict charge transport within the QD solid. This results in reduced sensitivity, slow response speed, and increased noise in photoconductive sensor devices [8,9,10,11,12,13,14,15,16,17,18,19,20,21,22,23,24,25,26,27,28,29,30,31,32,33,34,35]. Ligand exchange has been widely investigated as an effective strategy to overcome these limitations. Early approaches employed short organic molecules such as ethanedithiol and mercaptopropionic acid, followed by halide-based inorganic ligands that further improved carrier mobility and stability. Among various exchange methods, solution-phase ligand exchange offers the advantage of increasing quantum dot packing density prior to film formation, thereby enabling improved carrier transport compared to solid-state exchange methods. More recently, perovskite-related inorganic ligands have gained attention due to their favorable electronic properties and lattice compatibility with PbS QDs, which can further enhance charge transfer and interparticle coupling [36,37]. In this work, we report a high-sensitivity SWIR photodetector based on PbS QDs employing methylammonium lead iodide (MAPI) as an inorganic ligand introduced via solution-phase ligand exchange, where the effect of MAPI concentration on device performance is systematically investigated. To the best of our knowledge, a systematic investigation of MAPI ligand concentration effects in PbS QD-based SWIR photodetectors employing P3HT/ZnO transport layers has not yet been reported. Compared to conventional halide-based inorganic ligands, MAPI offers additional advantages owing to its perovskite crystal structure, which provides more complete surface passivation of PbS QDs and favorable band alignment for efficient charge extraction. Furthermore, its small lattice mismatch with PbS QDs and relatively high electrical conductivity enable the formation of a compact and electrically well-coupled QD thin film, making MAPI a particularly appropriate ligand choice for high-performance SWIR photodetectors. To further optimize charge extraction, poly(3-hexylthiophene-2,5-diyl) (P3HT) was incorporated as the hole-transport layer due to its well-matched valence band with PbS QDs, facilitating efficient hole extraction, while ZnO nanoparticles were employed as the electron-transport layer owing to their favorable conduction band alignment that promotes electron transport [38]. The resulting device structure exhibits significantly enhanced sensitivity, faster response speed, and improved signal-to-noise characteristics compared to devices based on organic-ligand-capped PbS QDs. These results demonstrate that MAPI-capped PbS QDs constitute a viable material platform for solution-processed, eye-safe SWIR photodetectors suitable for next-generation sensing applications.

## 2. Methods

### 2.1. Synthesis of Colloidal PbS QDs

**Figure 1:**
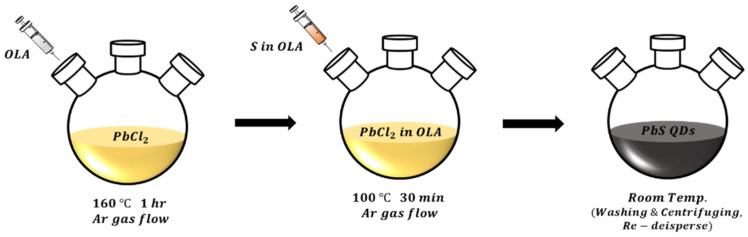
Schematic of the synthesis of PbS QDs.

Colloidal PbS quantum dots were prepared using a modified synthetic route adapted from previously reported procedures [39]. Initially, 0.1155 g of sulfur (S, Sigma-Aldrich, St. Louis, MO, USA) powder was combined with 2.4 mL of oleylamine (OLA, Sigma-Aldrich, St. Louis, MO, USA) in a three-neck flask and stirred for 30 min at room temperature under an argon atmosphere. Separately, 0.2781 g (1 mmol) of lead chloride (PbCl_2_, Sigma-Aldrich, St. Louis, MO, USA) was dissolved in 5 mL of OLA, stirred for 30 min at room temperature under Ar, and subsequently heated to 160 °C for 1 h. To eliminate impurities, the solution was degassed under vacuum at 120 °C for 15 min. Then, 225 μL of the pre-prepared S–OLA solution was swiftly injected into the PbCl_2_–OLA mixture and maintained at 100 °C under Ar to initiate QD formation. After a 30-min reaction, the mixture was cooled to room temperature. Methanol and toluene were then added at a 4:1:1 volume ratio for purification. The product was centrifuged at 4000 rpm for 5 min to collect the PbS QDs. Finally, the purified QDs were redispersed in hexane (20 mg/mL), yielding a stable PbS QD solution, as illustrated in Figure 1

### 2.2. Ligand Exchange of PbS QDs

**Figure 2:**
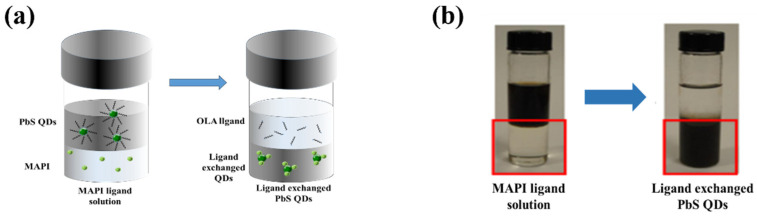
(**a**) Schematic, (**b**) experiments of MAPI ligand exchange process. Red squares indicate the transition of the PbS QD layer from the upper to the lower phase after ligand exchange.

Lead iodide (PbI_2_, Sigma-Aldrich, St. Louis, MO, USA) and methylammonium iodide (MAI, Sigma-Aldrich, St. Louis, MO, USA) were utilized as precursors for the methylammonium lead iodide (MAPI, Sigma-Aldrich, St. Louis, MO, USA) ligand exchange process. Initially, PbI_2_ and MAI were dissolved in N,N-dimethylformamide (DMF, Sigma-Aldrich, St. Louis, MO, USA) solvent at concentrations of 0.025, 0.05, and 0.1 mmol, respectively, to prepare the ligand exchange solution. Subsequently, the synthesized PbS QD solution was added to the ligand solution at a 1:1 volume ratio and stirred for 5 min to initiate the organic–inorganic ligand exchange reaction, followed by precipitation for 1 h. To eliminate unreacted or residual species, the resulting solution was washed with a mixture of hexane and the exchange solution in a 2:1 ratio, and this washing procedure was repeated three times to obtain high-purity QDs. Finally, toluene was added at a 1:1 ratio, and the MAPI-ligand-exchanged PbS QDs were collected by centrifugation. The purified MAPI-capped PbS QDs were then redispersed in butylamine, a polar solvent, at a concentration of 20 mg/mL to yield the final MAPI-capped PbS QD solution (Figure 2).

### 2.3. Device Fabrication

**Figure 3:**
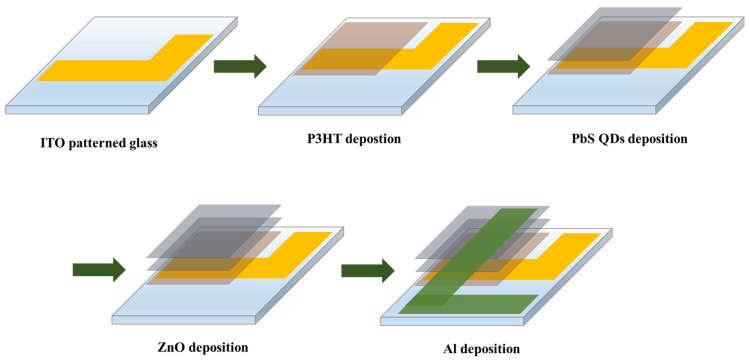
Schematic diagram of fabrication of SWIR photodetector based on PbS QDs.

A SWIR photodetector utilizing PbS QDs was fabricated by spin-coating a thin film onto an indium tin oxide (ITO)-patterned glass substrate. Before fabrication, the ITO-coated glass was sequentially cleaned with acetone, methanol, and isopropyl alcohol (IPA) to remove surface contaminants. As the hole transport layer, a conductive polymer solution of P3HT (20 mg/mL in chloroform) was prepared and spin-coated onto the cleaned substrate at 1500 rpm for 15 s, followed by annealing at 95 °C for 30 min to eliminate residual solvent. Subsequently, depending on the type of QD ligand, OLA-capped PbS QDs and MAPI-capped PbS QDs were separately spin-coated under identical conditions (1500 rpm, 15 s) to form the active sensing layer and then annealed at 95 °C for 30 min. A ZnO nanoparticle layer (20 mg/mL in ethanol) was then deposited by spin-coating at 1500 rpm for 30 s to serve as the electron transport layer, followed by annealing at 80 °C for 30 min. Finally, an aluminum electrode with a thickness of 150 nm was deposited by thermal evaporation, completing the fabrication of a photodetector with an active sensing area of 9 mm^2^ (Figure 3).

### 2.4. Sensor Measurement System

**Figure 4:**
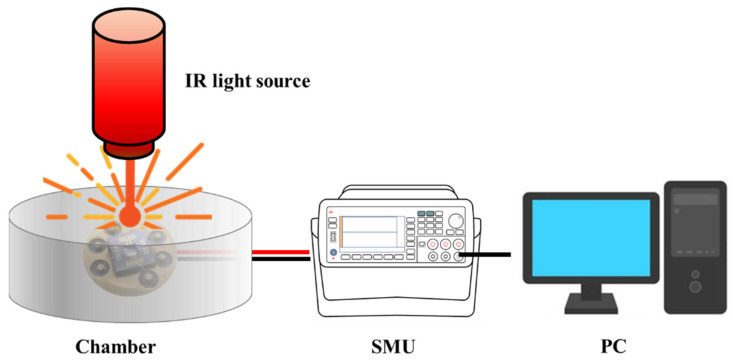
Schematic diagram of the measurement system.

To evaluate the electrical and optical performance of the fabricated SWIR photodetector, the variation in current upon IR light irradiation was measured. All experiments were carried out in a dark chamber to eliminate the influence of external light sources. The fabricated device was secured with a probe tip, and voltage was applied using a source meter unit (SMU, B2902A, Keysight Technologies, Santa Rosa, CA, USA) to measure current characteristics. An IR light source (SLS 202L/M, Thorlabs, Inc., Newton, NJ, USA) was positioned on the top side of the chamber to provide controlled illumination, as shown in Figure 4. The dark current (I_dark_), defined as the current measured without illumination, and the photocurrent (I_light_), defined as the current measured under illumination, were confirmed. By comparing I_dark_ and I_light_, the photocurrent-to-dark-current ratio (PDCR) of the SWIR photodetector was analyzed to confirm its light response characteristics. To exclude the contribution of visible wavelength light to the photoresponse, a longpass optical filter (FELH0700, cutoff wavelength: 700 nm, Thorlabs, Inc., Newton, NJ, USA) was placed between the light source and the device during all measurements. The incident optical power on the device active area was measured using a calibrated power meter (Thorlabs S401C) and determined to be approximately 1.067 μW.

## 3. Results

### 3.1. Characteristics of the Synthesized PbS QDs

During the solution-phase ligand exchange, the iodide anions (I^−^) from MAPI coordinate to undercoordinated Pb^2+^ sites on the PbS QD surface, replacing the long-chain oleylamine ligands. The methylammonium cations (MA^+^) do not directly passivate the surface but contribute to charge balance and colloidal stability of the exchanged QDs. This results in reduced interparticle spacing and elimination of insulating organic barriers, thereby enhancing carrier mobility within the QD film.

**Figure 5:**
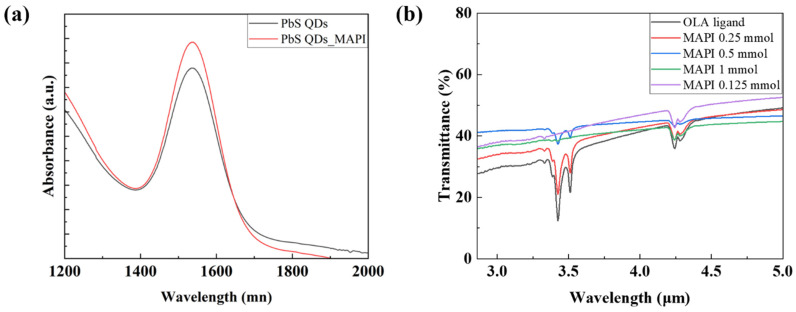
(**a**) Absorption spectra and (**b**) FT-IR analysis according to ligand exchange.

To verify the optical and chemical characteristics of the synthesized QDs, absorbance spectra and FT-IR analysis were conducted. As shown in Figure 5a, the absorption peak of the MAPI-capped PbS QDs appeared at 1550 nm, identical to that of the OLA-capped PbS QDs, with a full width at half maximum (FWHM) of approximately 92 nm. These results indicate that the absorption wavelength and FWHM of the PbS QDs were unaffected by the ligand exchange to the inorganic MAPI ligand, confirming that the MAPI-capped PbS QDs maintain stable optical properties and can selectively detect IR light around 1550 nm. This wavelength band is known to be eye-safe, suggesting that photodetectors fabricated using MAPI-capped PbS QDs can exhibit both high selectivity and responsivity while ensuring safety for human vision.

As shown in Figure 5b, FT-IR analysis was performed to verify successful ligand exchange through functional group characterization. A PbS QD solution (20 mg/mL) was spin-coated at 1000 rpm for 15 s onto a 10 × 10 mm^2^ glass substrate to form a thin film and then annealed at 95 °C for 30 min to prepare the measurement sample. The FT-IR spectra revealed that the characteristic C–H stretching peak in the 2800–3000 cm^−1^ region decreased as the MAPI concentration increased and completely disappeared when the MAPI concentration reached 1 mmol. This result confirms that the long-chain C–H bonds of the OLA organic ligand were replaced by the MAPI inorganic ligand, indicating a successful ligand exchange process. Additionally, a weak absorption peak observed near 2350 cm^−1^ was attributed to trace amounts of unremoved PbCl_2_ precursor remaining from the synthesis stage.

**Figure 6:**
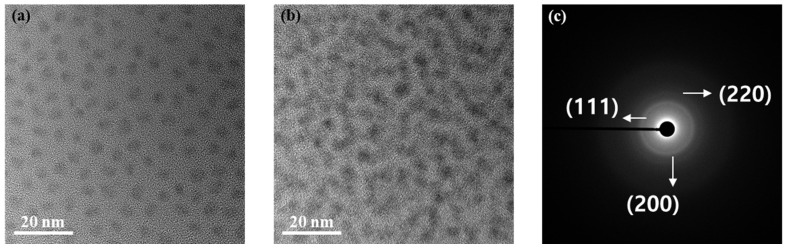
TEM image of PbS QDs according to (**a**) OLA ligand, (**b**) MAPI ligand, and (**c**) SAED characterization.

In addition, transmission electron microscopy (TEM) analysis was carried out to compare the surface density of the synthesized PbS QDs within the thin films (Figure 6). The TEM specimens were prepared by forming a thin layer of the synthesized PbS QDs on a mesh grid. The TEM observations demonstrated that the areal number density of the MAPI-exchanged PbS QDs was notably higher under identical imaging conditions. This increase is attributed to the substitution of the long-chain organic OLA ligands with shorter inorganic MAPI ligands, which reduced the interdot spacing between neighboring PbS QDs. As a result, a highly compact and uniform PbS QD thin film was formed, contributing to enhanced carrier transport and improved optoelectronic properties of the photodetector. Furthermore, the lattice fringe spacing of the quantum dots was analyzed using selected area electron diffraction (SAED), and both OLA- and MAPI-capped PbS QDs exhibited diffraction patterns consistent with XRD results, confirming that the crystallographic structure and phase purity of the PbS nanocrystals were maintained after ligand exchange.

### 3.2. Performance of the SWIR Photodetector

**Figure 7:**
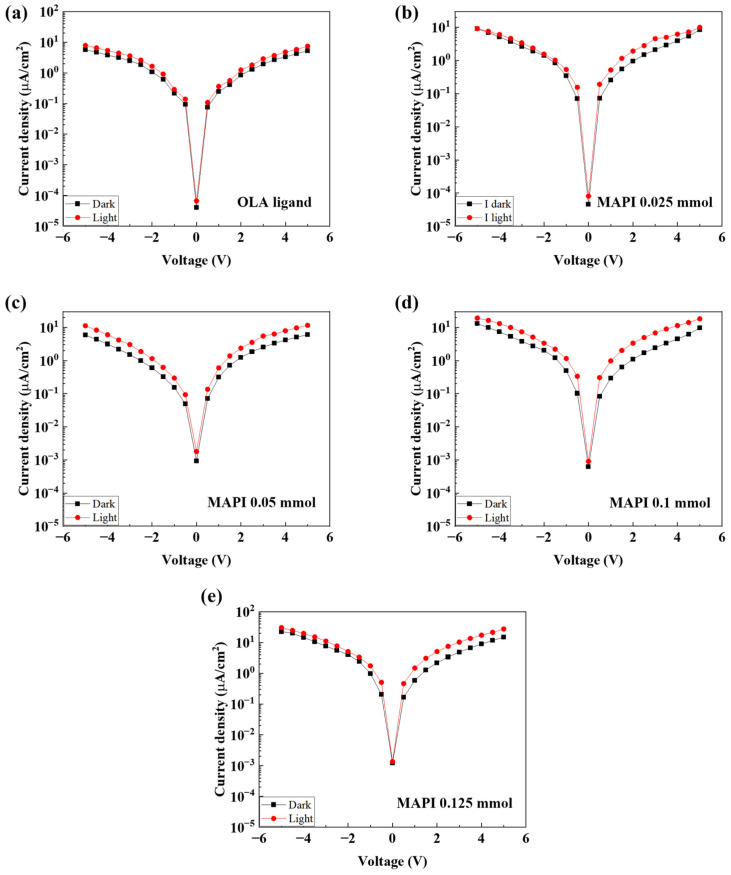
J–V characteristics according to light source irradiation. (**a**) OLA ligand, (**b**) MAPI 0.025 mmol, (**c**) MAPI 0.05 mmol, (**d**) MAPI 0.1 mmol, and (**e**) MAPI 0.125 mmol.

To investigate the performance characteristics of the fabricated SWIR photodetector based on PbS QDs, the device was mounted inside a dark chamber, and its photo response to the incident light source was evaluated. Initially, the current density–voltage (J–V) characteristics were measured to determine the on/off ratio of the SWIR photodetectors fabricated under different ligand conditions. The dark current density was obtained in the absence of illumination, while the photocurrent density was measured under light irradiation as the applied voltage was swept from −5 V to 5 V. As shown in Figure 7, the results revealed that the device exhibited the highest on/off ratio at an applied voltage of 3 V, indicating the maximum PDCR under this condition. At voltages exceeding 3 V, the on/off ratio decreased due to a disproportionate increase in dark current, which also resulted in higher power consumption. Therefore, 3 V was determined to be the optimal bias for subsequent measurements. Accordingly, all subsequent measurements and analyses were performed under the optimized operating voltage of 3 V to ensure consistent evaluation of the photodetector’s performance.

Next, the current response characteristics of the fabricated SWIR photodetector were analyzed under conditions with and without real-time light irradiation. The PbS QD-based photodetector operates on the photoconductivity principle, in which light absorption generates electron–hole pairs within the QD layer, leading to an increase in current. In the absence of illumination, the device exhibits dark current, representing its intrinsic electrical behavior, whereas under illumination, it exhibits photocurrent, which reflects the contribution of photo-generated carriers.

**Figure 8:**
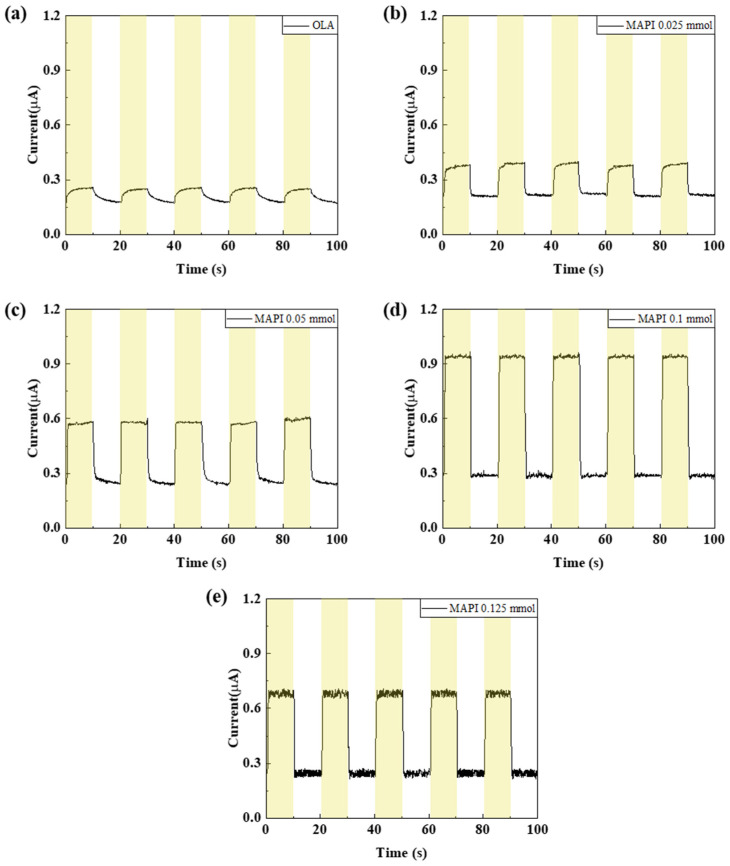
Real-time current characteristics according to light source irradiation. (**a**) OLA ligand, (**b**) MAPI 0.025 mmol, (**c**) MAPI 0.05 mmol, (**d**) MAPI 0.1 mmol, and (**e**) MAPI 0.125 mmol.

**Figure 9:**
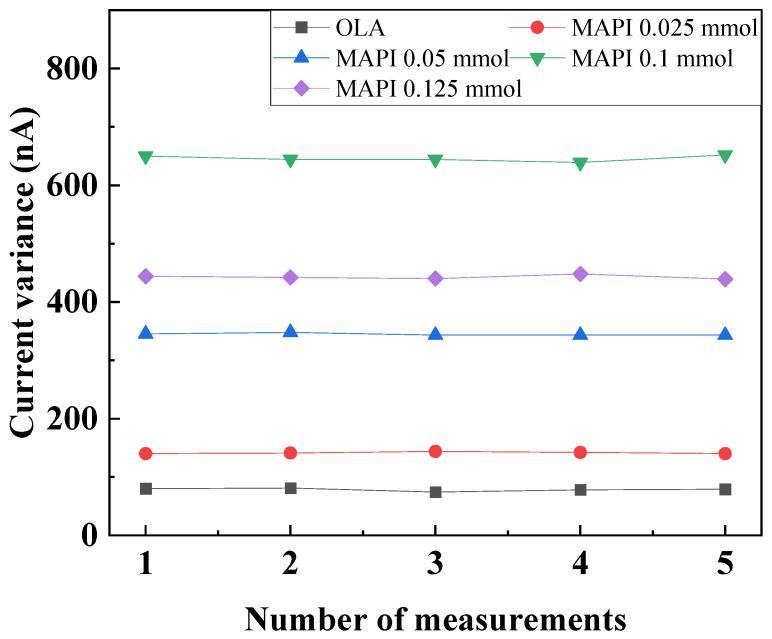
Current characteristics according to MAPI concentration.

As shown in Figure 8, the SWIR photodetector employing MAPI-capped PbS QDs exhibited a significantly higher current variation compared to that based on OLA-capped PbS QDs, with the maximum change observed at a MAPI concentration of 0.1 mmol. The corresponding current variance is summarized in Figure 9. For the OLA-capped PbS QD device, the current difference between dark and illuminated states reached approximately 60 nA, and the current response was unstable due to noticeable electrical noise. In contrast, the MAPI-capped PbS QD devices demonstrated dark-to-light current differences of 140 nA, 345 nA, 650 nA, and 442 nA, depending on MAPI concentration—corresponding to an enhancement of up to about 2.67 times compared to the OLA-capped device. Furthermore, the current response of the MAPI-based device was notably more stable, with reduced noise. This improvement can be attributed to the replacement of long insulating organic ligands (OLAs) with short inorganic MAPI ligands, which enhanced charge transport by reducing interparticle barriers and increasing QD film density per unit area. However, at an excessive MAPI concentration of 0.125 mmol, increased noise was observed, likely due to the presence of unreacted MAPI residues acting as impurities. To quantitatively compare the photoresponse, the PDCR of the SWIR photodetector was calculated using the following relation under an incident optical power of approximately 1.067 μW.

PDCR = I_light_/I_dark_, (1)

**Figure 10:**
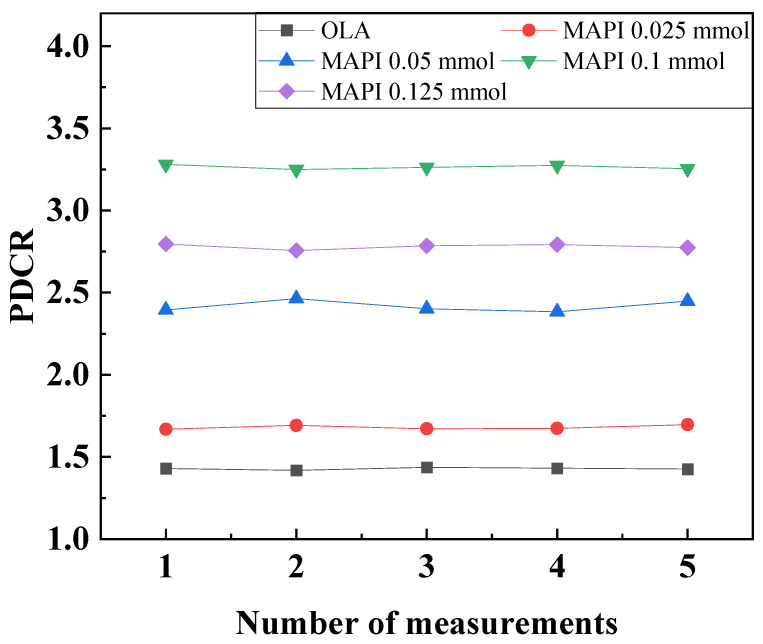
Photocurrent-to-dark-current ratio according to MAPI concentration.

As shown in Figure 10, the conventional OLA-capped PbS QD device exhibited an average PDCR of approximately 41%, whereas the MAPI-capped PbS QD device achieved an average PDCR of about 225%, representing an improvement of up to 5.48 times.

**Figure 11:**
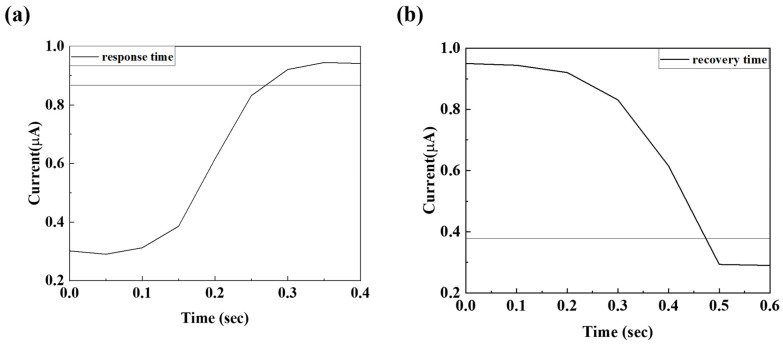
(**a**) Rise time and (**b**) fall time of the MAPI-ligand-exchanged SWIR photodetector.

The rise time (τr) and fall time (τf) are defined as the time required for the photocurrent to increase from 10% to 90% and decrease from 90% to 10% of its maximum value, respectively. As shown in Figure 11, the rise time improved markedly—from 4.15 s for the OLA-capped device to 0.26 s for the MAPI-capped device—corresponding to an enhancement of approximately 11.5 times. Similarly, the fall time improved from 4.15 s for the OLA-capped device to 0.45 s for the MAPI 0.1 mmol device, confirming the superior optoelectronic response enabled by MAPI ligand exchange.

The resistance and photoresponse time characteristics of all fabricated devices are summarized in Table 1. As the MAPI concentration increased from OLA to 0.1 mmol, R_dark_ decreased from 17.4 MΩ to 4.6 MΩ and both rise and fall times improved, which is attributed to reduced interdot spacing and enhanced carrier mobility enabled by the shorter MAPI ligands, as well as changes in the RC constant of the device. At 0.125 mmol, R_dark_ increased to 6.9 MΩ and the fall time slightly increased compared to the 0.1 mmol device, which is attributed to excess MAPI ligands disrupting the film uniformity and increasing interdot spacing, consistent with the increased noise observed in Figure 8e.

The external quantum efficiency (EQE) and responsivity [R(λ)] of the SWIR photodetector with the device structure ITO/P3HT/PbS QDs/ZnO/Al were characterized using a quantum yield spectrometer (Quantaurus-QY Plus, Hamamatsu Photonics, Hamamatsu, Japan). The EQE represents the ratio of the number of charge carriers (electrons and holes) successfully collected by the external circuit to the total number of incident photons on the device, thus reflecting how effectively the photogenerated carriers are extracted without undergoing recombination losses. Meanwhile, responsivity [R(λ)] indicates the magnitude of the photocurrent produced per unit optical power at a specific wavelength, essentially quantifying how sensitively the detector converts incoming light into electrical signals. Both EQE and R(λ) are key performance indicators that determine the overall efficiency and spectral selectivity of a photodetector. A high EQE value suggests efficient charge separation and transport within the active layer, while high responsivity demonstrates strong photoconversion capability, particularly important for low-intensity or broadband light detection. In this study, EQE measurements were carried out across the SWIR spectral range, and the corresponding responsivity was derived from the EQE spectrum using the following equation:

R(λ) = EQE × qλ/hc(2)

**Figure 12:**
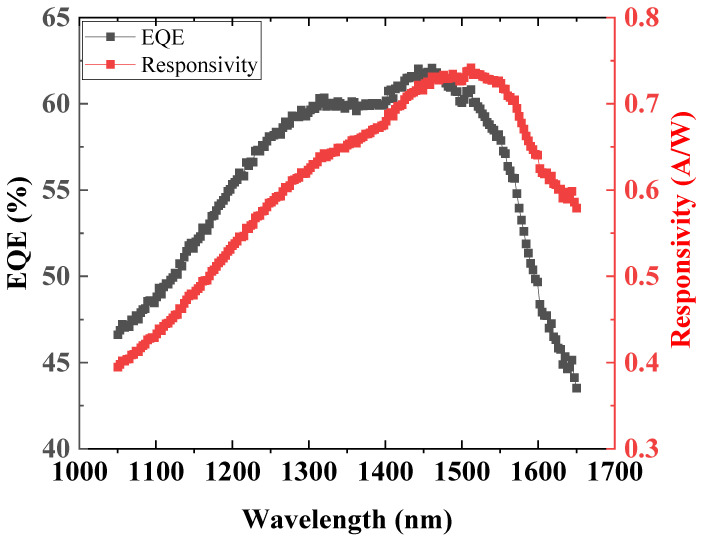
External quantum efficiency and responsivity of the MAPI-ligand-exchanged SWIR photodetector.

The parameters h, c, and λ denote Planck’s constant, the speed of light in vacuum, and the wavelength of the incident light, respectively, with the unit of hc/λ expressed in electron volts (eV). The EQE of the fabricated SWIR photodetector was measured across the spectral range of 1000–1650 nm, and the corresponding responsivity [R(λ)] was derived. As illustrated in Figure 12, the device exhibited outstanding photoresponse characteristics, achieving an EQE of approximately 62% within the 1400–1500 nm range. In the same spectral region, the responsivity reached a maximum value of 0.73 A/W, confirming the high photon-to-electron conversion efficiency and excellent optoelectronic performance of the fabricated SWIR photodetector.

**Figure 13:**
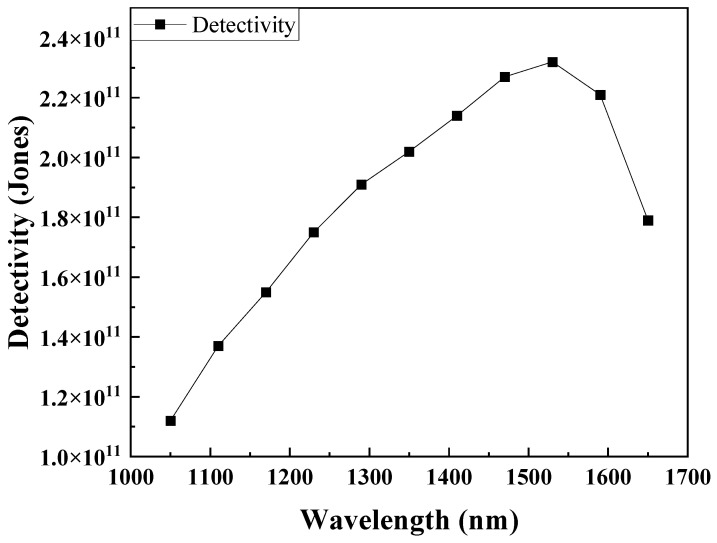
Detectivity of the MAPI-ligand-exchanged SWIR photodetector.

The specific detectivity (D*) was subsequently calculated using the measured R(λ) and the corresponding dark current under varying bias voltages. Detectivity is a critical figure of merit (FOM) for photodetectors, representing the inverse of the noise-equivalent power (NEP), which defines the minimum detectable optical power limited by intrinsic device noise. A higher D* value indicates superior PDCR, particularly advantageous for detecting weak optical signals near the noise threshold. In this analysis, it was assumed that the dominant noise component originated from the dark current shot noise under biased operating conditions. Accordingly, the noise-limited detectivity (D*) can be expressed by the following relation:

(3) $$D^{*}=\frac{R\left(\lambda \right)A^{1/2}}{{\left(2qI_{dark}\right)}^{1/2}}$$

where R(λ) denotes the wavelength-dependent photoresponsivity, q is the elementary charge, A represents the active area of the device, and I_dark_ corresponds to the dark current of the photodetector under reverse bias conditions. As shown in Figure 13, the fabricated SWIR photodetector exhibited relatively stable detectivity characteristics within the reverse bias range of several hundred millivolts, indicating that D* is not strongly bias-dependent in this regime. Moreover, the device demonstrated a high detectivity exceeding 2.26 × 10^11^ Jones across the long-wavelength spectral region of 1000–1400 nm, confirming its excellent PDCR and capability to detect weak infrared signals with minimal noise contribution. This result validates the effectiveness of the MAPI-capped PbS QDs structure in enhancing both carrier transport and signal-to-noise performance in SWIR photodetection applications. Table 2 reveals the performance comparison of different photodetectors within 5 years. The results show that the performance of the photodetector from this work had higher responsivity and detectivity. 

## 4. Conclusions

In this study, a solution-processed SWIR photodetector based on PbS quantum dots with inorganic MAPI ligands was successfully developed and systematically investigated. The conventional long-chain organic ligands of PbS QDs were replaced by methylammonium lead iodide through a solution-phase ligand exchange process, enabling the formation of a compact QD thin film with enhanced electrical coupling and charge transport properties. The MAPI-capped PbS QDs maintained stable optical absorption characteristics in the eye-safe SWIR region around 1550 nm, while exhibiting markedly improved photoconductive performance compared to their organic-ligand-capped counterparts. The fabricated photodetector demonstrated a high external quantum efficiency of 62%, a responsivity of 0.73 A/W, and a detectivity of 2.26 × 10^11^ Jones in the 1400–1500 nm wavelength range. In addition, the device showed significantly enhanced PDCR and a faster rise time, highlighting the effectiveness of inorganic ligand engineering for improving SWIR sensor performance. The improved optoelectronic characteristics are attributed to reduced interparticle spacing, increased quantum dot packing density, and more efficient carrier transport enabled by the MAPI ligand, as well as optimized charge extraction through the P3HT and ZnO transport layers. Systematic linear dynamic range (LDR) characterization was beyond the scope of the present experimental setup and will be investigated in future work using a dedicated 1550 nm monochromatic laser source with controlled optical power modulation. Owing to its simple device architecture, solution-based fabrication, and competitive sensing performance, the proposed MAPI-capped PbS QD photodetector offers strong potential for practical implementation in eye-safe LIDAR systems, optical sensing, and emerging autonomous and industrial monitoring technologies.

## Figures and Tables

**Table 1:** Resistance and photoresponse time characteristics of SWIR photodetectors with different MAPI ligand concentrations.

Ligand	R_dark_ (MΩ)	R_light_ (MΩ)	Rise Time (s)	Fall Time (s)
OLA	17.4	11.7	4.15	4.15
MAPI 0.025 mmol	7.9	3.7	0.30	0.40
MAPI 0.05 mmol	5.2	2.4	0.30	0.85
MAPI 0.1 mmol	4.6	1.6	0.26	0.45
MAPI 0.125 mmol	6.9	3.2	0.28	0.55

**Table 2:** Comparison of SWIR photodetector performance.

Wavelength (nm)	Responsivity (A/W)	Detectivity (Jones)	References
1540	0.264	1.47 × 10^11^	[40]
2100	0.385	1.5 × 10^11^	[41]
1550	0.97	2.21 × 10^10^	[42]
1310	0.26	1.1 × 10^11^	[43]
1550	0.73	2.26 × 10^11^	This work

## Data Availability

The original contributions presented in this study are included in the article. Further inquiries can be directed to the corresponding author.
